# Prevalence and Risk Factors Associated With Chronic Kidney Disease Among Patients Presenting at a Haemodialysis Unit in Dodoma, Tanzania

**DOI:** 10.24248/EAHRJ-D-16-00367

**Published:** 2018-04-01

**Authors:** Alfred J Meremo, Matobogolo B Masalu, Issa Sabi, David P Ngilangwa, Janet Kapinga, Rehema Tagalile, Mariam J Munyogwa, Masumbuko Y Mwashambwa

**Affiliations:** a School of Medicine and Dentistry, College of Health Sciences, The University of Dodoma, Dodoma, Tanzania; b Haemodialysis Unit, The University of Dodoma, Dodoma, Tanzania; c National Institute for Medical Research, Mbeya Medical Research Centre, Mbeya, Tanzania; d Amref Health Africa, Dar es Salaam, Tanzania

## Abstract

**Background:**

Chronic kidney disease (CKD) is a major public health problem worldwide, due to its epidemic proportions and the associated cardiovascular morbidity and mortality. However, data on the burden of CKD among patients attending hospitals in Tanzania are still limited. The aim of this study was to determine the prevalence and risk factors associated with CKD among patients presenting at the University of Dodoma (UDOM) haemodialysis unit in Tanzania.

**Methods:**

In this retrospective study, we reviewed data of 1,395 patients who presented at the UDOM haemodialysis unit from January 2013 to June 2015. Data were descriptively and inferentially analysed using Stata version 11.0.

**Results:**

From January 2013 to June 2015, a total of 1,395 patients presented at the UDOM haemodialysis unit with history of kidney disease. Of these patients, 1244 (89.2%) enrolled into this study, 651 (52.3%) of them were female. Almost two-thirds (n=792, 63.7%) of the patients were found to have CKD, 59.1% with an estimated glomerular filtration rate of <60 mL/min/1.73 m^2^. Among those who had CKD, 347 (43.8%) had hypertension, 241 (30.4%) had diabetic mellitus, 79 (10.0%) had chronic glomerulonephritis, 70 (8.8%) had hypertension and diabetes mellitus, 38 (4.8%) had HIV/AIDS, and 17 (2.1%) had hepatitis B. The median serum creatinine level was 222 lmol/L (interquartile range [IQR] 126 to 317), urea level was 14.5 mmol/L (IQR 5 to 24), hemoglobin was 11.0 g/dL (IQR 6.2 to 15.7), and body mass index was 27.1 kg/m^2^ (IQR 17.3 to 36.8). Obesity, diabetes mellitus, and systolic hypertension were associated with developing CKD (*P*<.001). A total of 116 patients received haemodialysis during the study period.

**Conclusion:**

CKD was common among patients presenting in our hospital and is associated with high cardiovascular risk. To that end, patients should be thoroughly evaluated to identify and correct causes of their kidney disease, and efforts should be put in place for early detection and screening as well as advocacy on risk factors for CKD development in Tanzania.

## INTRODUCTION

Chronic Kidney Disease (CKD) is found in 10% of the global population, and is considered a major public health problem worldwide, due to its epidemic proportions and its association with high cardiovascular risk.^1^ CKD is defined as abnormalities of kidney structure or function present or decreased estimate glomerular filtration rate (eGFR) for more than 3 months with implications for health, and classified into 5 stages according to the eGFR.^2–5^ The current recommendation is to use serum creatinine concentration to estimate the eGFR and transform it using the CKD Epidemiology Collaboration (CKD-EPI) equation.^5^

CKD is associated with age-related renal function decline accelerated in hypertension, diabetes, obesity and primary renal disorders.^6^ Although cardiovascular disease (CVD) is the primary cause of morbidity and mortality, CKD is regarded as an accelerator of CVD risk and an independent risk factor for CVD events.^7^ Also CKD severely affects patients' health, lifestyle and well-being, and quality of life^8^; it is at least 3 to 4 times more frequent in Africa than in developed countries.^9^ Despite high demand, very few patients in some African countries receive renal replacement therapy, mainly because of resource constraints.^10^

A study conducted between 2007 and 2010 in United States found that an estimated 14.0% of the U.S adult population had CKD.^11^ Although the causes vary, diabetes was the most common cause of CKD in the United States and is steadily increasing as the cause worldwide.^12^ In a study conducted in a hospital in Singapore, researchers found that 87.7% of the patients had moderate to severe CKD (stage 3 and above); the majority of the patients had dyslipidemia (92.8%), hypertension (89.3%) and diabetes mellitus (64.6%).^13^ In a study conducted in South Africa's black population, researchers found that primary hypertension occurred in 25.0% of the study population and was considered the cause of stage 5 CKD in 40.0% to 60.0% of these patients.^2^ In another study conducted in South Africa, the authors also reported hypertension as the leading cause in the adult population and the cause of chronic kidney failure in 21.0% of patients on renal replacement therapy registry.^9^ The prevalence of diabetic nephropathy is estimated to be 14.0% to 16.0% in South Africa, 23.8% in Zambia, 12.4% in Egypt, 9% in Sudan, and 6.1% in Ethiopia.^9^ In a study conducted in Nigeria, the overall prevalence of CKD was 18.8%. The researchers attributed the disease to hyper-tension (30.0%), diabetes mellitus (3.7%), obesity (14.6%), and haematuria (3.1%.), with age, female gender, systolic blood pressure, and diabetes mellitus as predictors of CKD.^14^

In a study of outpatients in North Western Tanzania, an alarmingly high prevalence (87.3%) of CKD was identified among adult patients attending diabetes mellitus clinic.^15^ In a community-based study conducted in Northern Tanzania, the overall prevalence of CKD was found to be 7%; and, of these patients, 19.3% had hypertension alone, 14.0% had both diabetes and hypertension, 7.0% had diabetes alone, 7.0% had HIV alone, and 3.5% had both HIV and hypertension. Nearly half (49.2%) of the cases of CKD were not associated with any of the measured risk factors of hypertension, diabetes, or HIV.^16^ Another hospital study conducted in North Western Tanzania, reported that hypertension-related diseases were the most common cause of hospital admission and CKD, and accounted for most deaths.^17^

While CKD is associated with high mortality and morbidity, limited data is available on the burden of CKD among patients attending hospitals in Tanzania, and, to date, the country has no national registry for CKD. Very few studies on prevalence of CKD have been reported in Tanzania, those that have been conducted have focused on patients either attending diabetic clinics or seeking care in communities.^15–17^ The aim of this study was to determine the prevalence and risk factors associated with CKD among patients presenting at the University of Dodoma (UDOM) haemodialysis unit in Dodoma, Tanzania.

## METHODS

### Study Design, Population, and Settings

Data for this retrospective study data were collected from patients who presented at the UDOM haemodialysis unit from January 2013 to June 2015. The UDOM hospital started its operations in 2007, when the university was officially launched, with the main aim of providing health-care services to the UDOM staff and students as well as the community of Dodoma. The hospital serves about 120,000 people, has a bed capacity of 100, and has specialist clinics conducted and run by medical specialists from the UDOM College of Health Sciences. In January 2013, the UDOM hospital launched its haemodialysis unit. The unit serves as a referral centre for the Singida, Morogoro, Iringa, Manyara, and Tabora regions, and for other regional referral hospitals in the central and other neighbouring zones, serving a total population of 20 million people.

All patients who required haemodialysis were treated at the unit, and all patients who presented at the haemodialysis unit but had no indications for haemodialysis were scheduled for routine medical follow up and continuous medical care.

### Data Collection and Laboratory Procedures

Patient medical record files of the 1,395 patients who presented at the UDOM haemodialysis unit from January 2013 to June 2015 were reviewed. Study data were obtained from handwritten medical records and then cross-checked with the electronic records. Any discrepancies were reviewed and verified to ensure the validity of data. The data were carefully reviewed and all patients with incomplete records were excluded. Information on patient sociodemographics, clinical characteristics, and relevant laboratory investigations were extracted. The study team reviewed information about age, gender, marital status, weight in kilograms, height in centimetres, clinical signs (oedema, anuria, hypertension), and laboratory data—including urinalysis, hepatitis panel, full blood picture, urea, creatinine, electrolytes, random blood glucose, and HIV test results—were recorded.

Body mass index (BMI) was calculated using the National Health Services (United Kingdom) BMI calculator, an eGFR was calculated using the CKD-EPI equation, and bedside isotope-dilution mass spectrometry-traceable Schwartz GFR calculator for children was used to stage patients.

Acute kidney injury was defined as an acute deterioration in renal excretory function, with a serum urea >10 mmol/L and/or a rise in serum creatinine (Scr) by ≥0.3 mg/dL, or a percentage increase in Scr of ≥50% from baseline using the Acute Kidney Injury Network criteria. End stage renal disease was defined as progressive CKD with eGFR ≤15 mL/min/1.73 m^2^ with or without other indications for haemodialysis. Outcome measures were CKD and its associated risk factors.

### Data Analysis

The de-identified data collected were entered into a computer using EpiData version 3.1 (CDC, Atlanta, USA) and analysed using Stata version 11.0 (StataCorp LLC, College Station, Texas, USA). Data were summarized as proportions and frequency tables for categorical variables. Depending on variable distribution, either mean with standard deviation or median with interquartile range were used to summarize continuous data. The correlation between the development of CKD and different patient parameters was determined by performing logistic regression analyses. Odds ratios (OR) were calculated to estimate the percentage change in risk of CKD development, and parameters with P values <.05 were considered statistically significant.

### Ethical Considerations

The study was approved by the UDOM research and publications ethics review board. Waiver of consent was approved by the committees as this retrospective study analysed only de-identified data.

## RESULTS

### Demographic and Other Characteristics of the Study Population

A total of 1,395 patients presented at the UDOM haemodialysis unit from January 2013 to June 2015. Of these patients, 151 (10.8%) were excluded from this study due to incomplete records, and the remaining 1,244 (89.2%) patients were enrolled in this study. The median age of the study population was 36 years (interquartile range [IQR] 6 to 88), over half (n=651, 52.3%) were female, more than half (n=704, 56.6%) were classified as obese, and about a third (n=434, 34.9%) reported having a history of smoking. A small percentage of the study population was living with HIV (n=57, 4.6%) or hepatitis B (n=26, 2.1%). Of the 1,244 patients enrolled, 792 (63.7%) were found to have CKD, 59.1% with an eGFR of <60 mL/min/1.73 m^2^ and 452 (36.3%) had no CKD after screening. A total of 116 patients received haemodialysis during study period, 32 (27.6%) of whom were found to have acute kidney injury (**Table 1**).

**TABLE 1.** Baseline Characteristics of Patients Who Presented at the University of Dodoma Haemodialysis Unit

Characteristic	Proportion (%) or Median [IQR]
Sex	
Male	593 (47.7)
Female	651 (52.3)
Age, in years	36 [6–88]
Marital status	
Never married	357 (28.7)
Married	479 (38.5)
Divorced	215 (17.3)
Widow	193 (15.5)
Smoking status (ever)	
No	810 (65.1)
Yes	434 (34.9)
Obesity (BMI) kg/m^2^	27.1 [17.3–36.8]
Haemoglobin (g/dL)	11.0 [6.2–15.7]
Serum creatinine level (μmol/L)	222 [126–317]
Urea level (mmol/L)	14.5 [5–24]
HIV status	
Positive	57 (4.5)
Negative	1187 (95.4)
Hepatitis	
Positive	26 (2.1)
Negative	1218 (97.9)
Chronic kidney disease	
No	452 (36.3)
Yes	792 (63.7)
Classification of stage of chronic kidney disease (n=792)	
Stage one	32 (4.0)
Stage two	292 (36.9)
Stage three	220 (27.7)
Stage four	164 (20.7)
Stage five	84 (10.6)

Abbreviations: BMI, body mass index; IQR, interquartile range.

### Causes of CKD Among Patients Who Presented at the Haemodialysis Unit

Of the 792 patients found to have CKD, 347 (43.8%) had hypertension, 241 (30.4%) had diabetes mellitus, 79 (10.0%) had chronic glomerulonephritis, and 70 (8.8%) had both hypertension and diabetes mellitus, while 38 (4.8%) and 17 (2.1%) were associated with HIV/AIDS and hepatitis B, respectively (**Table 2**).

**TABLE 2.** Causes of Chronic Kidney Disease Among Patients Who Presented at the Haemodialysis Unit

Medical Condition	Patients with CKD n (%)
Hypertension	347 (43.8%)
Diabetes mellitus	241 (30.4%)
Chronic glomerulonephritis	79 (10.0%)
Both hypertension and diabetes mellitus	70 (8.8%)
HIV/AIDS	38 (4.8%)
Hepatitis	17 (2.1%)

Abbreviation: CKD, chronic kidney disease.

### Risk Factors Associated With CKD Among Patients Who Presented at the Haemodialysis Unit

A total of 15 potential risk factors were identified after performing univariate logistic regression analyses. Backward elimination reduced this to 4 parameters. The potential predictors identified were gender, age, obesity, history of smoking, and causes of CKD. Gender, age, and smoking were found to be significant in univariate logistic regression analyses. However, this significance was lost after multivariate logistic regression analyses (**Table 3**).

**TABLE 3.** Adjusted Odds Ratios for Risk Factors Associated With Chronic Kidney Disease

Parameters	Adjusted OR (95% confidence interval)	*P* value
Gender		.516
Male	1.00	
Female	1.3 (1.1–1.8)	
Age		.135
<50 years	1.00	
≥50 years	1.8 (0.9–3.7)	
Smoking status (ever)		.763
No	1.00	
Yes	2.6 (1.6–4.3)	
Obesity		<.001
<30	1.00	
≥30	8.7 (3.5–15.9)	
Causes of CKD		
Hypertension (systolic)	7.5 (3.94–11.68)	<.001
Diabetes mellitus	9.23 (4.64–19.57)	<.001

* P <.05

Abbreviations: CKD, chronic kidney disease; OR, odds ratio.

Obesity (BMI ≥30) was predictive of CKD development (OR 8.7; 95% confidence interval [CI], 3.5 to 15.9; *P*<.001). In addition, the multivariate adjusted odds of CKD development were 9.2 times higher for a patient with diabetes mellitus (OR 9.23; 95% CI, 4.64 to 19.57; *P*<.001). Patients with systolic hypertension (OR 7.5; 95% CI, 3.94 to 11.68; *P*<.001) also had higher odds of developing CKD.

## DISCUSSION

This study evaluated the prevalence and risk factors associated with CKD at the UDOM hospital. A total of 792 (63.7%) patients enrolled in the study were found to have CKD, of whom 59.1% had an eGFR of <60 mL/min/1.73 m^2^. These findings are lower than an earlier study conducted by Janmohamed et al in North Western Tanzania, that showed the prevalence of CKD to be 80.0% among adults with diabetes mellitus, with nearly 25.0% having an eGFR of <60 mL/min/1.73 m^2^.^15^ These findings are also contrary with a community-based study conducted in Northern Tanzania, where the overall prevalence of CKD was found to be 7.0%.^16^ The vast differences in the findings could be attributed to the difference in study populations.

In a hospital study conducted in Singapore, researchers found that 87.7% of patients had moderate to severe CKD^13^; while in another hospital study conducted in Nigeria, found the overall prevalence to be 18.8%.^14^ CKD was at least 3 to 4 times more frequent in Africa than in developed countries.^9,11^ In a country like Tanzania, where there is no registry or advocacy for CKD prevention or early detection of kidney diseases, more effort is needed to fight the growing epidemic of noncommunicable diseases.

Of all the patients in our study who had CKD, 347 (43.8%) had hypertension, 241 (30.4%) had diabetic mellitus, 79 (10.0%) had chronic glomerulonephritis, and 70 (8.8%) had both hypertension and diabetes mellitus. Similar findings have been reported from other studies conducted in low- and middle-income countries, whereby hypertension has been reported to be the leading cause of CKD.^2,9,10,13,14^ However, these findings are contrary to a community-based study conducted in Northern Tanzania that showed that nearly half (49.2%) of the cases of CKD were not associated with any of the measured risk factors of hypertension, diabetes, or HIV.^16^ This discrepancy calls for more research into other causes of CKD, including the use of local herbs and medicines, which is very rampant in this area where this study was conducted. Different findings have also been reported from the United States and other developed countries, whereby diabetes mellitus has been reported as the leading cause of CKD and is steadily increasing as the cause worldwide.^4,11,12,18^

Our study found that 704 (56.6%) patients were found to be obese and 434 (34.9%) patients reported having had a history of smoking. Similar findings have been reported from other studies whereby obesity and positive history of smoking were reported as risk factors for CKD.^14,19–21^ In this study obesity (BMI ≥30), diabetes mellitus and systolic hypertension were strongly associated with development of CKD (*P*<.001). Similar findings have been reported from other studies.^6,14,19–21^

### Limitations and Strengths

This study was conducted in a hospital in central part of Tanzania and, as such, the results are limited to patients in the central part of Tanzania. Despite the limited generalizability, this information could provide insight to strategies for improving the management of patients who present with CKD in hospitals. The strengths of this study were that our hospital uses both paper-based and electronic medical records, which allowed the recording and collection of patients' health related information in real time. The study data were obtained from handwritten medical records and then cross checked with the electronic records. Any discrepancies were reviewed and verified to ensure the validity of data.

## CONCLUSION

CKD was common among patients presenting in our hospital and is associated with high cardiovascular risk. To that end, patients should be thoroughly evaluated to identify and correct the causes of their disease. As CKD is regarded as an accelerator of cardiovascular risks and an independent risk factor for cardiovascular events and eventually death, efforts should be put in place for early detection and screening as well as advocacy on risk factors for CKD development in Tanzania.
